# Cystatin C as a Renal Biomarker in Infants with Congenital Anomalies of the Kidney and Urinary Tract (CAKUT): A Systematic Review

**DOI:** 10.3390/diagnostics16081115

**Published:** 2026-04-08

**Authors:** Mihaela Dobre, Ana Maria Cristina Jura, Ramona Stroescu, Daniela Eugenia Popescu, Vlad Laurentiu David

**Affiliations:** 1 Ph.D. School, Faculty of Medicine, “Victor Babeș” University of Medicine and Pharmacy, Eftimie Murgu Sq. No. 2, 300041 Timisoara, Romania; 2 Department of Neonatology, Clinical Hospital of Obstetrics and Gynecology “Dumitru Popescu”, Franyo Zoltan 6, 300014 Timisoara, Romania; 3 Department of Obstetrics and Gynecology, “Victor Babeş” University of Medicine and Pharmacy, Eftimie Murgu Sq. No. 2, 300041 Timisoara, Romania; 4 Medici’s MedLife Hospital Timișoara, Ciprian Porumbescu Street No. 9, 300236 Timisoara, Romania; 5 Department XI of Pediatrics-1st Pediatric Discipline, Center for Research on Growth and Developmental Disorders in Children, “Victor Babeș” University of Medicine and Pharmacy, Eftimie Murgu Sq. No. 2, 300041 Timisoara, Romania; 6 4th Pediatric Clinic, ‘Louis Țurcanu’ Children’s Clinical and Emergency Hospital, Iosif Nemoianu 2, 300011 Timisoara, Romania; 7 Department of Pediatric Surgery and Orthopedics, “Victor Babeş” University of Medicine and Pharmacy, Eftimie Murgu Sq. No. 2, 300041 Timisoara, Romania; david.vlad@umft.ro

**Keywords:** acute kidney injury (AKI), diagnosis, renal function, neonatal intensive care, biomarkers, therapeutic strategies, preterm newborns, chronic kidney disease

## Abstract

**Background**: The evaluation of renal function in neonates is challenging due to maternal creatinine transfer, reduced muscle mass, and non-steady-state physiology. Cystatin C emerged as a promising biomarker for assessing neonatal glomerular filtration rate. This review summarizes evidence from studies evaluating serum and urine cystatin C in healthy neonates and high-risk groups, including preterm newborns, neonates with acute kidney injury, and those with congenital kidney and urinary tract defects. **Methods**: Twenty studies were included and qualitatively synthesized following PRISMA guidelines. **Results**: In the included studies, serum cystatin C exhibited consistent postnatal patterns independent of maternal influence and showed a strong correlation with gestational age and renal development. Cystatin C enabled earlier detection of renal dysfunction compared to serum creatinine, especially in preterm infants and critically ill neonates. In babies with congenital renal abnormalities, cystatin C levels were associated with disease severity and clinical outcomes, while the cystatin C-based estimated glomerular filtration rate surpassed creatinine-based estimations. Urinary cystatin C correlated with tubular damage and increased risk of chronic kidney disease during follow-up. **Conclusions**: Cystatin C is a reliable biomarker for evaluating neonatal renal function, although further standardization and validation are required for clinical implementation.

## 1. Introduction

Cystatin C has emerged as a promising biomarker for assessing renal function in newborns, especially those with congenital abnormalities of the kidney and urinary tract (CAKUT). The precise evaluation of renal function in neonates has historically depended on serum creatinine; however, creatinine is unreliable in early life as it crosses the placenta, mirrors maternal renal function for up to 48–72 h after delivery, and is influenced by muscle mass, gestational age, bilirubin concentrations, and tubular secretion [1,2,3,4]. These limitations are particularly concerning in premature infants, with reduced muscle mass and underdeveloped tubular function, as well as in neonates with suspected structural renal disease, where prompt detection of compromised glomerular filtration rate (GFR) is critical for prognosis and clinical management [5,6,7]. Cystatin C, a 13 kDa cysteine protease inhibitor produced at a constant rate by all nucleated cells, is well suited for evaluating newborn renal function. It is readily filtered by the glomerulus, completely reabsorbed and metabolized in the proximal tubules, and is neither secreted nor reintroduced into circulation [8]. Cystatin C does not cross the placenta, and therefore neonatal levels reflect the infant’s intrinsic renal function [9]. Nephrogenesis is generally completed between 35 and 36 weeks of gestation. Therefore, preterm infants are born with an incomplete nephron complement and reduced baseline glomerular filtration capacity. In this context, elevated cystatin C levels may reflect both physiological immaturity and reduced nephron number rather than pathological renal injury alone. Consequently, gestational age must be considered when interpreting cystatin C values [10,11]. Cystatin C exhibits impressively stable developmental patterns across gestational ages, with a predictable decline as renal perfusion and filtration enhance [12]. This stability allows clinicians to more easily detect aberrant renal function patterns compared to creatinine, especially in preterm infants and individuals at risk for acute kidney injury (AKI).

CAKUT accounts for 40–60% of pediatric chronic kidney disease (CKD) globally. Prenatal renal dysfunction in CAKUT can result in oligohydramnios, pulmonary hypoplasia, and increased risk of perinatal respiratory distress. Early identification of renal dysfunction is essential for prognosis and clinical management. Numerous investigations have shown that cystatin C is closely correlated with the severity of CAKUT, exceeds creatinine in the early detection of renal impairment, and may forecast survival in severe instances [13].

Furthermore, newborn acute kidney injury is widely acknowledged as a significant factor in the long-term risk of chronic kidney disease, even in infants without anatomical anomalies [14]. Cystatin C increases prior to creatinine in acute kidney injury, offering a vital opportunity for early management. In addition to glomerular biomarkers, urine cystatin C has surfaced as a sensitive indicator of proximal tubular damage and may offer further understanding of chronic kidney disease progression in previously preterm infants [15].

The limits of creatinine and the increasing evidence that cystatin C more correctly reflects both renal function and prognosis suggest that integrating cystatin C into neonatal renal evaluation methods is a significant achievement in pediatric nephrology. This review consolidates existing research about cystatin C in healthy newborns, populations with congenital anomalies of the kidney and urinary tract (CAKUT), premature infants, and individuals experiencing acute kidney injury (AKI) or at risk of chronic kidney disease (CKD). It also assesses analytical factors, physiological interpretations, and practical applications for NICU practitioners.

## 2. Materials and Methods

This systematic review was conducted and reported in accordance with the Preferred Reporting Items for Systematic Reviews and Meta-Analyses (PRISMA) 2020 statement, tailored for a biomarker-centric examination of neonatal renal physiology and clinical outcomes.

### 2.1. Review Design and Rationale

In light of the growing acknowledgement of cystatin C as a superior marker of neonatal glomerular filtration rate (GFR) compared to serum creatinine, a systematic review methodology was used to assess its efficacy in various neonatal cohorts. The included studies involved healthy term neonates, preterm infants, newborns with congenital abnormalities of the kidney and urinary tract (CAKUT), neonates undergoing acute kidney injury (AKI), and infants predisposed to chronic kidney disease (CKD). The review assessed urine cystatin C with serum cystatin C as a marker of tubular integrity and a potential predictor of long-term renal prognosis. The analytical approach was guided by known reviews of pediatric renal biomarkers, with unique modifications to accommodate newborn physiological variability and biomarker-specific factors.

### 2.2. Eligibility Criteria

#### 2.2.1. Inclusion Criteria

- Neonates (0–28 days) or preterm infants up to 44 weeks postmenstrual age.
- Studies reporting serum and/or urinary cystatin C.
- Studies evaluating renal function, AKI, CAKUT, or CKD outcomes.
- Original research studies.

#### 2.2.2. Exclusion Criteria

- Studies without cystatin C measurements.
- Non-neonatal populations.
- Non-renal outcomes.
- Reviews, editorials, letters, or animal studies.

#### 2.2.3. Identification and Screening of Studies

A comprehensive literature search was performed across PubMed/MEDLINE, Embase, Wiley Online Library, Scopus, and the Cochrane Library to identify relevant studies published between January 1990 and December 2025. The search strategy used combinations of Medical Subject Headings (MeSH) terms and free-text keywords, including “cystatin C,” “neonate,” “newborn,” “infant, newborn,” “preterm,” “premature infant,” “acute kidney injury,” “AKI,” “congenital anomalies of the kidney and urinary tract,” “CAKUT,” “renal function,” “glomerular filtration rate,” and “chronic kidney disease.” Boolean operators were applied using the structure (“cystatin C” AND (neonate OR newborn OR preterm OR premature infant)) AND (renal function OR glomerular filtration rate OR acute kidney injury OR CAKUT OR chronic kidney disease). The initial search yielded approximately 1240 records after removal of duplicates. Title/abstract and full-text screening were performed independently by two reviewers, with disagreements resolved by consensus to exclude non-neonatal studies, non-renal outcomes, and articles without cystatin C data. Following this screening, 148 full-text articles were assessed for eligibility. After detailed full-text review, studies were excluded due to lack of neonatal cystatin C measurements, absence of relevant renal outcomes, or insufficient methodological detail. Disagreements between reviewers were resolved by consensus, resulting in 20 studies included in the final qualitative synthesis.

The full electronic search strategy for PubMed is provided in Supplementary Table S1.

### 2.3. Data Extraction Process

A standardized data extraction template was used for each qualifying study. Extracted variables encompassed study design, characteristics of the neonatal population including gestational age and presence of CAKUT or AKI risk factors, sample size, timing of biomarker assessments, cystatin C assay methodology, reference values and temporal trends, correlations with serum creatinine, urine output, imaging findings, or clinical outcomes, definitions of AKI utilized, and documented renal outcomes such as survival, requirement for dialysis, or progression to CKD. This approach is consistent with previously described frameworks for neonatal AKI and biomarker assessment.

### 2.4. Risk of Bias and Methodological Quality Assessment

Methodological quality and certainty of evidence were assessed qualitatively in accordance with PRISMA recommendations, using GRADE-informed principles, as standard diagnostic accuracy tools were not fully applicable due to heterogeneity in study design and outcomes. Standard instruments like QUADAS-2 or the Newcastle–Ottawa Scale [16,17] were not fully applicable due to heterogeneity in study design and outcome reporting, a recognized limitation of neonatal biomarker research. The evaluation concentrated on the precision of the neonatal population definition, timing and frequency of cystatin C measurements, analytical techniques and assay calibration, the suitability of reference intervals, the application of validated AKI definitions such as modified KDIGO criteria [18], the sufficiency of sample size, and the thoroughness of follow-up regarding long-term renal outcomes. Special emphasis was placed on research concerning premature infants or CAKUT, where the interpretation of biomarkers is significantly affected by developmental factors.

### 2.5. Outcome Measures

The main outcomes of interest were represented by blood cystatin C levels in healthy term and preterm neonates, focusing on gestational age-specific reference values and postnatal temporal patterns. Further primary outcomes included the efficacy of cystatin C in neonates with congenital anomalies of the kidney and urinary tract (CAKUT), specifically its capacity to predict mortality, necessity for clinical or surgical intervention, and severity of renal dysplasia, alongside the diagnostic performance of cystatin C in neonatal acute kidney injury, which involved sensitivity, timing of elevation, and overall accuracy. Urinary cystatin C was assessed as a primary outcome, serving as an indicator of proximal tubular damage and a possible predictor of chronic kidney disease risk. Secondary outcomes encompassed comparisons of cystatin C and serum creatinine in various neonatal settings, assessment of cystatin C-derived estimated glomerular filtration rate (eGFR) equations, and examination of analytical and methodological variables affecting cystatin C measurement and interpretation.

### 2.6. Synthesis of Evidence

The findings were synthesized using a narrative integrative technique, as advised for diverse biomarker literature. Studies were categorized thematically instead of undergoing meta-analysis due to variations in assay methodologies, date of sample collection, and demographic factors. Physiological alterations at various gestational ages were assessed in conjunction with pathological patterns to yield a comprehensive analysis of cystatin C in newborn renal evaluation. This methodology corresponds with modern paradigms for synthesizing neonatal AKI biomarkers, where meta-analysis frequently encounters limitations due to heterogeneity.

### 2.7. Protocol and Registration

The review protocol was not registered, and no a priori protocol was prepared. The completed PRISMA 2020 checklist is provided in the Supplementary Materials [19].

## 3. Results

**Figure 1:**
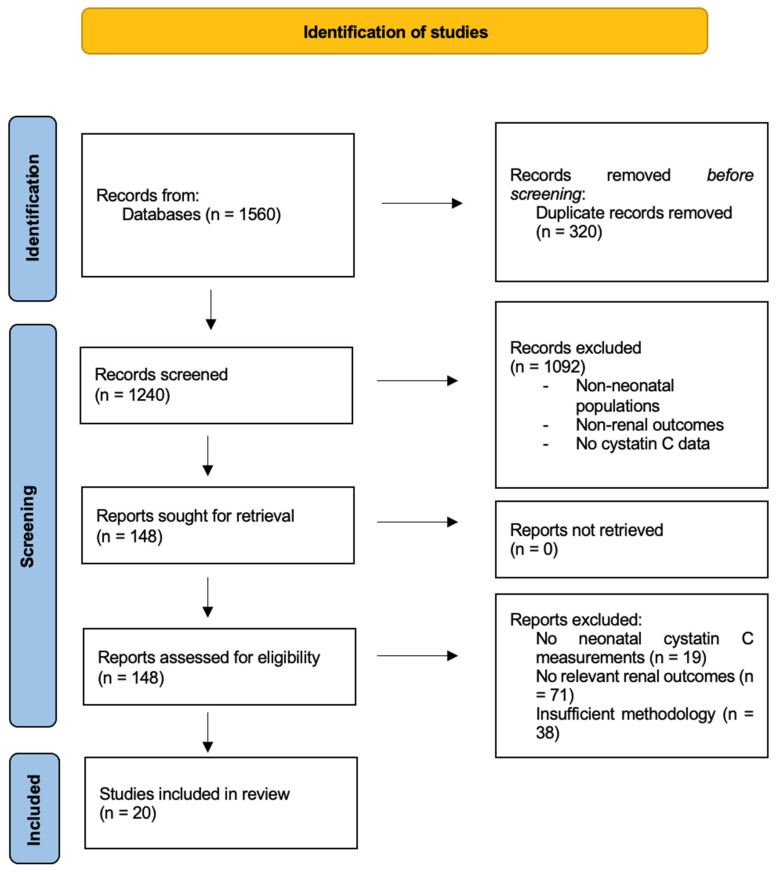
PRISMA flow diagram of study selection for our review.

A total of 20 neonatal renal biomarker studies met the inclusion criteria after detailed screening (Figure 1). These were synthesized with the broader literature to interpret cystatin C behavior across neonatal populations, including healthy term infants, preterm infants, newborns with congenital anomalies of the kidney and urinary tract (CAKUT), neonates with acute kidney injury (AKI), and infants at risk for chronic kidney disease (CKD). Cystatin C performance was also compared with serum creatinine and urinary tubular biomarkers. The following sections present the integrated findings.

### 3.1. Included Studies

This review encompasses 20 studies, shown in Table 1, that assessed cystatin C in diverse neonatal demographics and clinical scenarios, yielding consistent results about its physiological validity and therapeutic applicability. The included studies were predominantly single-center observational studies with heterogenous methodologies, sample sizes, and outcome definitions.

### 3.2. Serum Cystatin C in Healthy Neonates

Serum cystatin C concentrations in healthy term neonates demonstrate consistent patterns across multiple studies. Immediately after birth, term infants typically exhibit concentrations ranging between 1.6 and 2.8 mg/L, reflecting the combined influence of low glomerular filtration rate (GFR) and physiologic neonatal renal immaturity [2,21,22]. Unlike serum creatinine, which is heavily influenced by maternal renal function and placental transfer [39], neonatal cystatin C levels represent intrinsic renal function from the moment of birth. This distinction is critical for accurate early renal assessment.

Over the first postnatal week, cystatin C levels decline along with increasing renal perfusion, maturation of glomerular hemodynamics, and structural nephron stabilization [40,41]. Studies consistently show that cystatin C decreases by approximately 0.2–0.4 mg/L per day during the first week, stabilizing at 1.0–1.6 mg/L by day seven in healthy term newborns [42]. This predictable downward trend was consistently observed across studies and enables early identification of deviations suggestive of renal pathology [20,21,22,23,25,26,32].

Unlike creatinine, which may paradoxically rise in the first 48 h regardless of renal injury due to maternal washout [43], cystatin C follows a unidirectional, physiologically governed trajectory. Consequently, clinicians can reliably interpret abnormal cystatin C patterns—particularly plateaued or rising values—as indicative of impaired GFR or early renal dysfunction.

### 3.3. Serum Cystatin C in Preterm Infants

Preterm infants consistently exhibit higher serum cystatin C concentrations than term infants, a finding attributed to limited nephron endowment, ongoing nephrogenesis, lower baseline renal blood flow, and structural immaturity of glomeruli and tubules [44,45]. Extremely preterm infants (<28 weeks of gestation) may present with cystatin C levels exceeding 2.5–3.5 mg/L at birth, substantially higher than term infants [46]. This reflects both limited filtration capacity and the biological immaturity of the neonatal kidney [21,24,28,33].

The trajectory of cystatin C decline in preterm infants is slower and more variable than in term infants. Serial measurements indicate that cystatin C may remain elevated for several weeks in very low birth weight (VLBW) or extremely low birth weight (ELBW) neonates [47]. These prolonged elevations correlate with delayed postnatal maturation of GFR, consistent with classical physiological studies of preterm renal function [48,49,50]. Several investigators have demonstrated that cystatin C more reliably reflects gestational age-dependent renal maturation than creatinine, which may be misleadingly low in severely growth-restricted or sarcopenic premature infants [51,52].

Importantly, cystatin C differentiates between physiological immaturity and pathological renal dysfunction more clearly than creatinine. In preterm infants, a lack of cystatin C decline over the first week or a secondary rise often precedes clinically diagnosed AKI, suggesting its potential as an early biomarker for monitoring renal vulnerability in this population [53,54,55].

### 3.4. Serum Cystatin C in Congenital Anomalies of the Kidney and Urinary Tract (CAKUT)

#### 3.4.1. Pathophysiological Relevance of Cystatin C in CAKUT

CAKUT represents a spectrum of structural anomalies—including renal dysplasia, hydronephrosis, ureteropelvic junction obstruction, posterior urethral valves, and renal agenesis—that impair nephron development, compromise fetal urine output, and alter renal function at birth [56,57,58]. These defects often manifest as oligohydramnios, pulmonary hypoplasia, and varying degrees of renal insufficiency [3,27]. Accurate early postnatal assessment is critical for predicting outcomes, determining urgency of intervention, and guiding longitudinal nephrology follow-up.

#### 3.4.2. Cystatin C Levels Correlate with CAKUT Severity

Across studies, cystatin C demonstrates stronger correlations with CAKUT severity than creatinine [34,36]. Severely affected infants—particularly those with bilateral renal dysplasia or lower urinary tract obstruction—often show cystatin C values exceeding 3.5–4.5 mg/L at birth, markedly higher than both healthy term infants and mildly affected CAKUT patients [30,36,59]. These elevated levels reflect reduced nephron mass, compromised filtration surface area, and impaired renal perfusion.

The discriminatory power of cystatin C is evident in its ability to distinguish between mild hydronephrosis (with near-normal cystatin C values) and severe bilateral renal dysplasia or posterior urethral valves (with markedly elevated values) [60]. Creatinine, by contrast, frequently fails to capture these differences in the first days of life due to maternal influence and delayed kinetics [61].

#### 3.4.3. Prognostic Implications

Elevated cystatin C at birth is strongly associated with adverse clinical outcomes in CAKUT, including need for surgical intervention, respiratory compromise from pulmonary hypoplasia, and early mortality [62,63]. Infants with cystatin C levels > 4 mg/L in the immediate postnatal period are substantially more likely to require dialysis, ventilatory support, or transfer to tertiary care nephrology centers.

Cystatin C trajectories during the first postnatal week provide important prognostic insight in neonates with CAKUT. Declining cystatin C concentrations suggest preserved renal adaptive capacity and functional reserve, whereas persistently stable levels are more consistent with chronic impairment related to underlying renal dysplasia. In contrast, rising cystatin C values indicate worsening renal function and may reflect evolving urinary tract obstruction, superimposed acute kidney injury, or severe structural renal disease. These dynamic patterns closely align with the known pathophysiology of CAKUT and offer superior prognostic discrimination compared with creatinine-based assessments [64,65].

### 3.5. Cystatin C in Neonatal Acute Kidney Injury (AKI)

#### 3.5.1. Early Rise in Cystatin C Compared to Creatinine

A key finding in neonatal AKI research is the earlier and more prominent rise in cystatin C in response to hypoxic, ischemic, or nephrotoxic injury. Cystatin C levels typically increase within 12–24 h of decreased filtration, whereas creatinine may take 24–72 h to rise due to its slow accumulation and large maternal reservoir [66].

This early rise confers significant clinical advantages, enabling timely interventions—such as fluid optimization, avoidance of nephrotoxins, and hemodynamic support—which may reduce kidney injury progression [29,30,31].

#### 3.5.2. Sensitivity and Specificity

Several neonatal studies demonstrate higher diagnostic sensitivity and specificity for cystatin C in early AKI compared with creatinine [67,68]. A rise of ≥0.3 mg/L in cystatin C has been shown to predict AKI by KDIGO criteria with moderate-to-high accuracy, even before changes in urine output become apparent or creatinine rises.

#### 3.5.3. Utility in High-Risk Neonatal Populations

Cystatin C demonstrates superior predictive value in neonatal populations at high risk for acute kidney injury, including infants with hypoxic–ischemic encephalopathy undergoing therapeutic hypothermia, those exposed to nephrotoxic medications such as aminoglycosides, nonsteroidal anti-inflammatory drugs, or angiotensin-converting enzyme inhibitors, infants supported with extracorporeal membrane oxygenation, and neonates with hemodynamic instability or sepsis. In these high-risk settings, elevations in cystatin C frequently precede overt clinical manifestations of AKI by several days, highlighting its role as an early-warning biomarker that enables timely preventive and therapeutic interventions [69,70,71].

### 3.6. Urinary Cystatin C as a Biomarker of Tubular Injury

While serum cystatin C reflects GFR, urinary cystatin C serves as a marker of proximal tubular integrity [35,37,38]. Typically, cystatin C is completely reabsorbed and catabolized in the proximal tubule; thus, detectable urinary concentrations signal tubular dysfunction [72].

Studies in preterm infants have demonstrated that elevated urinary cystatin C concentrations are associated with prolonged mechanical ventilation, systemic inflammatory states, and exposure to nephrotoxic medications, reflecting underlying tubular stress or injury. Importantly, higher urinary cystatin C levels have also been linked to lower glomerular filtration rate at follow-up and an increased risk of long-term chronic kidney disease, supporting its value as an early indicator of adverse renal outcomes. Collectively, these findings position urinary cystatin C as a promising biomarker for detecting subclinical tubular injury and for mapping early chronic kidney disease trajectories in vulnerable neonatal populations [73].

Limited data are available regarding the simultaneous assessment of serum and urinary cystatin C in neonates. However, available evidence suggests that these measurements provide complementary information, with serum cystatin C reflecting glomerular filtration and urinary cystatin C indicating tubular injury [74]. Combined assessment may improve early detection and risk stratification in high-risk neonatal populations.

### 3.7. Comparison with Other Renal Biomarkers

In addition to cystatin C, other biomarkers such as neutrophil gelatinase-associated lipocalin (NGAL) and kidney injury molecule-1 (KIM-1) have been investigated in neonatal renal disease. NGAL is a sensitive early marker of acute kidney injury; however, its specificity is limited, as it may increase in inflammatory conditions. KIM-1 reflects proximal tubular injury but does not provide direct information about glomerular filtration [75,76].

In contrast, cystatin C directly reflects glomerular filtration rate and is independent of maternal influence, making it particularly valuable in neonates. In CAKUT, cystatin C provides more reliable assessment of renal function compared to tubular biomarkers alone. Therefore, while NGAL and KIM-1 may complement cystatin C, cystatin C remains the preferred biomarker for evaluating overall renal function and disease severity [77].

### 3.8. Cystatin C and Long-Term Renal Outcomes (CKD Risk)

Cumulative evidence suggests that abnormalities in serum or urinary cystatin C during the neonatal period may predict later renal dysfunction. Infants with persistent elevations in cystatin C beyond the first week—particularly preterm infants or infants with CAKUT—demonstrate higher risk for reduced GFR, hypertension, albuminuria, and CKD in childhood [78].

Nephron number, renal perfusion, and early injury synergistically determine CKD trajectory. Because cystatin C accurately captures all three dimensions—nephron mass, filtration capacity, and tubular injury—it offers a more comprehensive early indicator of CKD risk than creatinine or urine output.

## 4. Discussion

Serum cystatin C exhibited a consistent early-life trajectory, often elevated at birth with a predictable decrease over the initial week to month, thus confirming its utility as a maturation-adjusted filtration measure in neonates [20,21,22,23,24,32]. As described by Cataldi et al., this pattern aligns with extensive evidence indicating that cystatin C does not traverse the placenta, so reflecting neonatal rather than maternal renal function, which addresses a significant limitation of blood creatinine immediately postpartum [26,79]. The persistent observation that preterm infants exhibit elevated cystatin C levels and a protracted postnatal decline compared to term infants corresponds with developmental nephrology research associating prematurity with diminished nephron endowment, modified renal maturation, and an augmented risk of chronic kidney disease throughout life [47,80,81]. Interpretation of cystatin C in preterm infants must consider ongoing nephrogenesis, which continues until approximately 35–36 weeks of gestation. Elevated levels in this population may reflect physiological immaturity rather than intrinsic renal injury. Our review reinforces the clinical assertion that the interpretation of cystatin C should be tailored to gestational and postnatal age, rather than relying on a singular neonatal “normal range” [20,21,22,23,24,32].

In the NICU and high-risk neonatal populations, the research corresponds with the extensive AKI biomarker literature, indicating that cystatin C frequently increases prior to serum creatinine in the progression of kidney injury, hence enhancing the promptness of identification [3,27,34,36]. Specifically, as described by Elmas et al. preterm infants with respiratory distress syndrome and term neonates after perinatal asphyxia or hypoxic–ischemic injury exhibited earlier elevations of cystatin C compared to creatinine, aligning with the established delay in creatinine kinetics and the non-steady-state conditions characteristic of early neonatal life [34,36]. In the prospective study by Kandasamy et al., serum cystatin C identified impaired renal function earlier than serum creatinine in critically ill neonates, supporting its use in high-risk clinical settings [27]. These findings align with infant AKI conceptual frameworks advocating for standardized definitions (modified KDIGO) and the prompt identification of injury to facilitate nephrotoxin avoidance and hemodynamic optimization [3,82,83]. While creatinine-based definitions of acute kidney injury (AKI) are the norm, accumulating research indicates that cystatin C may serve as a risk-enrichment or early-warning biomarker in situations where timely intervention is critical and creatinine measurements are delayed [83].

In CAKUT, our analysis endorses cystatin C as a predictor of severity, as described by Tomotaky et al. [29] and a prognostic factor when assessed in cord blood or early postnatal life [29,30]. This guidance aligns with the extensive CAKUT and pediatric nephrology literature, highlighting that early functional evaluation can enhance imaging-based phenotyping, especially in renal dysplasia, where creatinine levels may under-represent severity due to maternal influence and reduced neonatal muscle mass [26,29,30,79]. The diagnostic accuracy study comparing cystatin C–based eGFR with creatinine-based eGFR in CAKUT corroborates existing nephrology data that cystatin C, whether used independently or in conjunction with creatinine, enhances GFR estimation efficacy compared to creatinine alone [30,84]. These accumulating data substantiate a CAKUT strategy wherein cystatin C is tested early (in cord blood and/or during the first week) and thereafter monitored serially to guide the intensity of surveillance and the time of nephrology intervention [29,30].

The studies presented connect early tubular injury to subsequent chronic kidney disease risk in extremely preterm newborns, providing further detail beyond filtration markers alone [35,37,38]. In extremely low birth weight infants, Askenazi et al. showed that elevated urinary cystatin C during the first two weeks of life was associated with tubular injury and increased risk of subsequent chronic kidney disease [35]. This aligns with modern conceptions of neonatal kidney disease as a continuum, wherein subclinical tubular injury may precede overt acute renal injury or diminished estimated glomerular filtration rate [35,37,38]. The extensive cohort connecting neonatal urinary biomarkers, such as urinary cystatin C, to diminished eGFR and CKD at follow-up offers significant longitudinal evidence for the inclusion of urinary biomarkers in the risk classification of extremely low gestational age neonates [38]. These findings further support the recognized epidemiological link between prematurity and chronic kidney disease (CKD) in the long run, highlighting the potential benefit of incorporating cystatin C (serum ± urine) into the follow-up protocols for NICU graduates [45,47,81]. Future research must emphasize assay standardization, clinically relevant cutoffs, and prospective protocols to evaluate whether cystatin C–guided management (nephrotoxin stewardship bundles and customized monitoring) decreases AKI incidence and enhances long-term renal outcomes [38,82].

### 4.1. Limitations of the Review

Although there is an increasing amount of evidence indicating that cystatin C is a more effective biomarker for newborn renal function, many limitations within the current literature must be recognized. The majority of existing studies are observational and conducted at single centers, exhibiting variability in assay techniques, scheduling of measurements, and definitions of outcomes, with a limited number offering long-term renal follow-up. However, the uniformity of results across various neonatal cohorts indicates significant clinical applicability, especially in high-risk environments such as prematurity, congenital renal and urinary tract anomalies, and perinatal hypoxic–ischemic injury, where creatinine-based evaluations are unreliable.

### 4.2. Future Perspectives

Integrating cystatin C into initial renal assessment may facilitate the prompt detection of kidney injury, enhance risk classification, and allow for more precise nephroprotective interventions in the newborn critical care unit. Future multicenter studies utilizing standardized assays, gestational age-specific reference intervals, and prospective assessments of cystatin C-guided clinical interventions are essential to establish actionable thresholds and elucidate its role in averting the progression from neonatal kidney injury to chronic kidney disease. This review recognizes cystatin C as a physiologically relevant and therapeutically significant biomarker capable of enhancing both short- and long-term renal outcomes in neonates. Prospective studies evaluating cystatin C-guided clinical pathways are now required to determine whether earlier detection translates into reduced AKI incidence and improved long-term renal outcomes.

## 5. Conclusions

Cystatin C is a reliable biomarker for renal function in neonates, and offers advantages over serum creatinine, particularly in early life and in high-risk populations. It may improve early diagnosis and risk stratification; however, further validation in large prospective studies is required before routine clinical implementation.

## Figures and Tables

**Table 1:** Summary of studies included in the review.

Study/Year	Population	Design	*N*	Biomarker (s)	Timing of Measurement	Key Outcomes	Comments/Limitations
**Ferreira Novo et al. [20]**	Healthy term neonates	Prospective longitudinal	82	Serum cystatin C	Birth, days 3–28	Predictable postnatal decline; independent of maternal creatinine	Single-center study
**Finney et al. [21]**	Term and preterm neonates	Prospective reference study	97	Serum cystatin C, serum creatinine	Birth to day 10	Gestational age specific reference ranges; cystatin C more reliable than creatinine	Few extremely preterm infants
**Harmoinen et al. [22]**	Term and preterm neonates	Prospective	84	Serum cystatin C	Birth to day 5	Higher cystatin C in preterm infants; correlates with renal maturation	No long-term outcomes
**Strevens et al. [23]**	Healthy term neonates	Prospective	50	Serum cystatin C	Birth to day 7	Decline parallels postnatal GFR maturation	No renal pathology
**Treiber et al. [24]**	Term and preterm neonates	Prospective methodological study	80	Serum cystatin C	Birth and early neonatal period	Developed and validated a cystatin C—based formula for estimating GFR in newborns	Single-center cohort; limited external validation
**Renganathan et al. [25]**	Term and preterm neonates	Prospective	68	Serum cystatin C	Birth to 1 month	Demonstrated gestational age—dependent cystatin C trajectories during the first postnatal month	Single-center study
**Cataldi et al. [26]**	Healthy term neonates	Prospective	45	Serum cystatin C	Cord blood	Demonstrated lack of placental transfer	Small cohort
**Kandasamy et al. [27]**	NICU neonates	Prospective	58	Serum cystatin C	First week of life	Earlier renal dysfunction detection than creatinine	Mixed clinical diagnoses
**Zaffanello et al. [28]**	Neonates and children	Observational	70	Serum cystatin C	Neonatal period	Reliable marker independent of muscle mass	Mixed pediatric population
**Tomotaki et al.** [29]	Neonates with CAKUT	Prospective	61	Serum cystatin C	Cord blood	Elevated cystatin C predicted early mortality	Short follow-up
**Parvex et al. [30]**	Neonates with CAKUT	Prospective	49	Serum cystatin C	Birth to day 7	Correlated with renal dysplasia severity	No long-term outcomes
**Steflea et al. [31]**	Infants with CAKUT	Diagnostic accuracy study	92	Cystatin C—based eGFR, creatinine-based eGFR	Neonatal period	Cystatin C—eGFR superior to creatinine	Single assay platform
**Dorum et al. [32]**	Term and preterm neonates	Prospective observational study	90	Serum cystatin C	Birth to early neonatal period	Established gestational-age specific serum cystatin C reference values	Single center; no long-term renal outcomes
**Guignard et al. [33]**	Preterm neonates	Physiologic cohort	44	Serum cystatin C, serum creatinine	Birth to day 14	Demonstrated creatinine unreliability in early life	Not outcome-focused
**Elmas AT et al. [34]**	Preterm neonates with RDS	Prospective observational study	60	Serum and urinary cystatin C	First 72 h of life	Elevated serum cystatin C levels predicted development of AKI before serum creatinine	Single center study; limited sample size
**Askenazi et al. [35]**	Extremely low birth weight infants	Prospective	120	Urinary cystatin C	Days 1–14	Predicted tubular injury and CKD risk	Urine collection variability
**Refat NH et al. [36]**	Neonates with HIE	Prospective observational study	80	Serum cystatin C, serum creatinine	First 72 h of life	Serum cystatin C rose significantly earlier than creatinine; cystatin C demonstrated higher sensitivity for early AKI prediction following perinatal asphyxia	Single center study; short term follow-up
**Selewski et al. [3]**	NICU neonates	Cohort	250	Serum cystatin C, serum creatinine	Daily	Improved AKI detection and prognosis	No urinary biomarkers
**Koralkar et al. [37]**	Preterm infants	Prospective follow-up	72	Serum cystatin C	Neonatal period and 2-year follow-up	Neonatal cystatin C predicted CKD traits	Underpowered for CKD endpoints
**Hingorani SR et al. [38]**	Extremely low birth weight infants	Prospective cohort	327	Urinary cystatin C, NGAL, IL-18, KIM-1	Neonatal period and longitudinal follow-up	Elevated urinary cystatin C associated with reduced eGFR and development of chronic kidney disease at follow-up	Biomarkers not measured serially in all infants; CKD outcomes assessed later in childhood

## Data Availability

The original contributions presented in this study are included in the article. Further inquiries can be directed to the corresponding author.

## Supplementary Materials

The following supporting information can be downloaded at: https://www.mdpi.com/article/10.3390/diagnostics16081115/s1, Table S1. Search Strategies Used for Literature Identification. File S1. PRISMA 2020 Checklist [19].

## Abbreviations

The following abbreviations are used in this manuscript:

AKI	Acute Kidney Injury
CAKUT	Congenital Anomalies of the Kidney and Urinary Tract
CKD	Chronic Kidney Disease
eGFR	Estimated Glomerular Filtration Rate
ELBW	Extremely Low Birth Weight
GA	Gestational Age
GFR	Glomerular Filtration Rate
HIE	Hypoxic–Ischemic Encephalopathy
IL-18	Interleukin-18
KDIGO	Kidney Disease: Improving Global Outcomes
KIM-1	Kidney Injury Molecule-1
NGAL	Neutrophil Gelatinase-Associated Lipocalin
NICU	Neonatal Intensive Care Unit
PMA	Postmenstrual Age
RBF	Renal Blood Flow
RDS	Respiratory Distress Syndrome
VLBW	Very Low Birth Weight
