# Supplementary material for: Cystatin C as a Renal Biomarker in Infants with Congenital Anomalies of the Kidney and Urinary Tract (CAKUT): A Systematic Review
Source: Diagnostics (Basel). 2026 Apr 8;16(8):1115. doi: 10.3390/diagnostics16081115 (PMC13114961; doi:10.3390/diagnostics16081115)
Supplement: Supplementary file 1 [file diagnostics-16-01115-s001.zip › Supplementary Table S1.docx]

**Supplementary materials**

**Supplementary Table S1. Search Strategies Used for Literature Identification**

| **Database** | **Search Strategy** | **Restrictions** |
| --- | --- | --- |
| **PubMed/MEDLINE** | (“cystatin C”[MeSH Terms] OR “cystatin C”[Title/Abstract]) AND (“infant, newborn”[MeSH Terms] OR neonate*[Title/Abstract] OR newborn*[Title/Abstract] OR preterm[Title/Abstract] OR premature infant*[Title/Abstract]) AND (“renal function”[Title/Abstract] OR “kidney function”[Title/Abstract] OR “glomerular filtration rate”[MeSH Terms] OR GFR[Title/Abstract] OR “acute kidney injury”[MeSH Terms] OR AKI[Title/Abstract] OR “congenital anomalies of the kidney and urinary tract”[Title/Abstract] OR CAKUT[Title/Abstract] OR “chronic kidney disease”[MeSH Terms] OR CKD[Title/Abstract]) | Human studies; no language restrictions; January 1990 to March 2025 |
| **Embase** | ('cystatin c'/exp OR 'cystatin c':ti,ab) AND ('newborn'/exp OR 'infant, newborn'/exp OR neonate*:ti,ab OR newborn*:ti,ab OR 'premature infant'/exp OR preterm:ti,ab) AND ('renal function'/exp OR 'kidney function':ti,ab OR 'glomerular filtration rate'/exp OR gfr:ti,ab OR 'acute kidney injury'/exp OR aki:ti,ab OR 'congenital anomalies of the kidney and urinary tract':ti,ab OR cakut:ti,ab OR 'chronic kidney disease'/exp OR ckd:ti,ab) | Human studies; January 1990 to March 2025 |
| **Scopus** | TITLE-ABS-KEY("cystatin C") AND TITLE-ABS-KEY(neonate* OR newborn* OR "infant, newborn" OR preterm OR "premature infant*") AND TITLE-ABS-KEY("renal function" OR "kidney function" OR "glomerular filtration rate" OR GFR OR "acute kidney injury" OR AKI OR "congenital anomalies of the kidney and urinary tract" OR CAKUT OR "chronic kidney disease" OR CKD) | Publications from 1990 to 2025 |
| **Wiley Online Library** | ("cystatin C") AND (neonate* OR newborn* OR preterm OR "premature infant") AND ("renal function" OR "glomerular filtration rate" OR "acute kidney injury" OR CAKUT OR "chronic kidney disease") | All years; human studies |
| **Cochrane Library** | (“cystatin C”) AND (neonate* OR newborn* OR preterm OR premature) AND (renal OR kidney OR AKI OR CAKUT OR CKD) | All years |
